# Heavy Metal Depuration Steps for *Gracilaria chilensis* in Outdoor Culture Systems

**DOI:** 10.3390/molecules27206832

**Published:** 2022-10-12

**Authors:** Jorge Rivas, Florentina Piña, Matías Araya, Nicolás Latorre-Padilla, Benjamín Pinilla-Rojas, Sofía Caroca, Francisca C. Bronfman, Loretto Contreras-Porcia

**Affiliations:** 1 Departamento de Ecología y Biodiversidad, Facultad de Ciencias de la Vida, Universidad Andres Bello, Santiago 8370251, Chile; 2 Centro de Investigación Marina Quintay (CIMARQ), Facultad de Ciencias de la Vida, Universidad Andres, Bello, Quintay, Valparaíso 2531015, Chile; 3 Center of Applied Ecology and Sustainability (CAPES), Santiago 8331150, Chile; 4 Instituto Milenio en Socio-Ecología Costera (SECOS), Santiago 8370251, Chile; 5 Programa de Doctorado en Biotecnología, Facultad de Ciencias de la Vida, Universidad Andres Bello, Santiago 8370251, Chile; 6 Institute of Biomedical Science (ICB), Faculty of Medicine, Universidad Andres Bello, Echaurren 183, Santiago 8320000, Chile

**Keywords:** depuration rate, *Gracilaria chilensis*, outdoor culture, purification, red seaweed, sustainability, seaweed food

## Abstract

Seaweed aquaculture is affected by natural and anthropogenic stressors, which put the biomass productivity of the cultures at risk. Seaweed biomass for commercial purposes, principally in pharmaceutical and/or nutraceutical applications, needs to be free of pollutants; therefore, controlled cultures have relevance in regulating the quality of biomass. The aim of this work was to demonstrate the successful utilization of controlled outdoor cultures to remove excess heavy metal accumulation in *Gracilaria chilensis*, an important commercial seaweed farming model. Specifically, we designed a simple and operational heavy metal depuration protocol, utilizing seawater and tap water removal, which permitted the concentration reduction of 10 heavy metals, including As, Cu, and Cd but not Zn, from the biomass at 7 days of culture. The percentage of depuration of the heavy metals ranged from 32 to 92% at 7 days, which was maintained throughout 21 days of culture. During the culture period, the monitored physicochemical parameters (temperature, salinity, and dissolved oxygen, among others) remained stable, with an increase in the daily growth rate (DGR% d^−1^) of the biomass recorded after 14 days of culture. Consequently, the experimental setup was successful for heavy metal depuration, which highlights the importance of controlled outdoor cultures as important tools of sustainability.

## 1. Introduction

Seaweed has been present in the human diet for many years and has been part of Asian cuisine culture for almost 400 years [1]. Currently, in addition to providing nutrition to humans, there is an increasing interest in its role in supporting human health, although almost 99% of algae production is dedicated to thickening and gelling agents for the food and pharmaceutical industries [2,3].

By 2020, out of a total of 35.07 million tons of aquatic seaweed produced globally, cultivated seaweed accounted for 87.8% of the total, but this high percentage was derived from only a small number of Asian countries, mainly China, Indonesia, the Republic of Korea, and the Philippines. Commonly cultivated seaweed species include *Laminaria japonica* (12.4 million tons), *Eucheuma* spp (8.1 million tons), *Gracilaria* spp (5.2 million tons), *Undaria pinnatifida* (2.8 million tons), and *Porphyra* spp (2.2 million tons) [4]. Chile is the main producer of seaweed in the West, with a production of 222,000 tons of seaweed, but this production depends mainly on the exploitation of natural populations. Additionally, around 60,000 tons of Chile’s production corresponds to *Gracilaria chilensis*, commonly known as “Pelillo” [5]. In Chile, *Gracilaria chilensis* C.J. Bird, McLachlan and E.C. Oliveira is distributed between Antofagasta (24° S) and Chiloé (43° S), with its southernmost population located in “Raúl Marín Balmaceda” port (43°46′ S) [6,7]. The extraction of *G. chilensis* from natural populations began in the mid-1970s and collapsed in the 1980s due to overexploitation. Its use as a food and a medicinal herb is long-standing, and although the “Pelillo” has great nutritional potential, it is mostly exploited for the extraction of agar and exported as dried algae [8,9]. Red algae such as *G. chilensis* are an excellent source of healthy essential fatty acids and other phytochemicals with bioactivities including antidiabetic, anti-inflammatory, antioxidant, and anti-neurodegenerative effects [2,10,11,12]. Recently, our laboratory reported that Gracilex^®^, an oily extract derived from “Pelillo”, can reduce metabolic alterations (increases in basal glucose and insulin levels) in mice fed a high-fat diet [13]. Thus, the development of a nutraceutical derived from *G. chilensis* offers an opportunity to find other uses for this alga to diversify the productive matrix of the country, increasing the added value of *G. chilensis* biomass. Thus, studies addressing the innocuity of the biomass used for nutraceuticals are required to expand this industry.

*G. chilensis* has a three-stage sexual life cycle characterized by an isomorphic alternation of generations [14]. In situ cultivation is based on vegetative propagation [15,16] or conducted with spores attached on ropes that are then transferred to the sea [17]. Thus, a decrease in genetic variability associated with overexploitation is expected, which may increase the susceptibility of algae to pests and epiphytes under monoculture conditions.

Seaweed farming may be affected by natural stressors (climate change, storms, and pests, among others) or anthropogenic stressors (heavy metal pollution, petroleum spills, and domestic and industrial wastes), which put the productivity and quality of the cultured biomass at risk [18,19]. In this context, controlled cultures (outdoor or indoor cultures) are useful for reducing unwanted variables that could affect the cultivars. In fact, controlled cultures have been used extensively to determine the tolerances of target species before starting mariculture programs, e.g., [20,21], and for strengthening the early production stages of the available biomass. Outdoor and indoor seaweed cultivation to produce a high biomass yield, e.g., [22], depend on several factors, for example, the type of cultivation, temperature, adequate irradiation, and nutrient concentration, among others, which must be balanced in order to promote an efficient biomass production [23,24]. Outdoor controlled cultures have a high yield potential; the control and mechanization of their main operations influence the uptake and assimilation of nutrients as well as the productivity of the culture [25,26]. Despite this, cultivation systems such as outdoor cultures that can mix the conditions of farmed seaweed cultivation and the development of the initial stages of growth can generate a relatively simple, productive, and ecofriendly seaweed farming system.

At present, it is necessary to determine the causes of pollution in coastal zones, principally by heavy metals, before seaweed farming. The term “heavy metals” is commonly used in the literature to refer to metals and metalloids associated with environmental pollution, toxicity, and adverse effects on biota and has been defined in various ways, mostly in terms of density, relative atomic mass, and atomic number [27]. Heavy metals are considered harmful and non-degradable pollutants. They are ubiquitous in natural environments, and these metals enter marine ecosystems mainly through atmospheric deposition, overland runoff, and industrial and domestic activities; they are mainly stored in abiotic components (i.e., sediment and seawater), which can be bioaccumulated by the organisms and biomagnified along the trophic webs [28,29,30].

It has been determined that seaweeds accumulate metals through a two-stage process that begins with rapid and reversible physicochemical adsorption on the algal surface, followed by a slower, metabolically arranged intracellular uptake [31,32], triggering diverse physiological alterations [33,34,35]. The cell wall biomolecules of seaweeds provide sites to which metals bind quickly; indeed, several species are considered efficient materials for heavy metal biosorption [36,37]. The seaweed biomass for commercial use, principally in pharmaceutical or nutraceutical applications, needs to be free of pollutants; therefore, the controlled cultures take on an important relevance in regulating the quality of the biomass. In this context, the aim of this work was to demonstrate the successful utilization of controlled outdoor cultures to remove excess heavy metal accumulation in *Gracilaria chilensis* as an important commercial seaweed farming model.

## 2. Results

### 2.1. Heavy Metal Bioconcentration in Gracilaria chilensis Biomass

**Figure 1:**
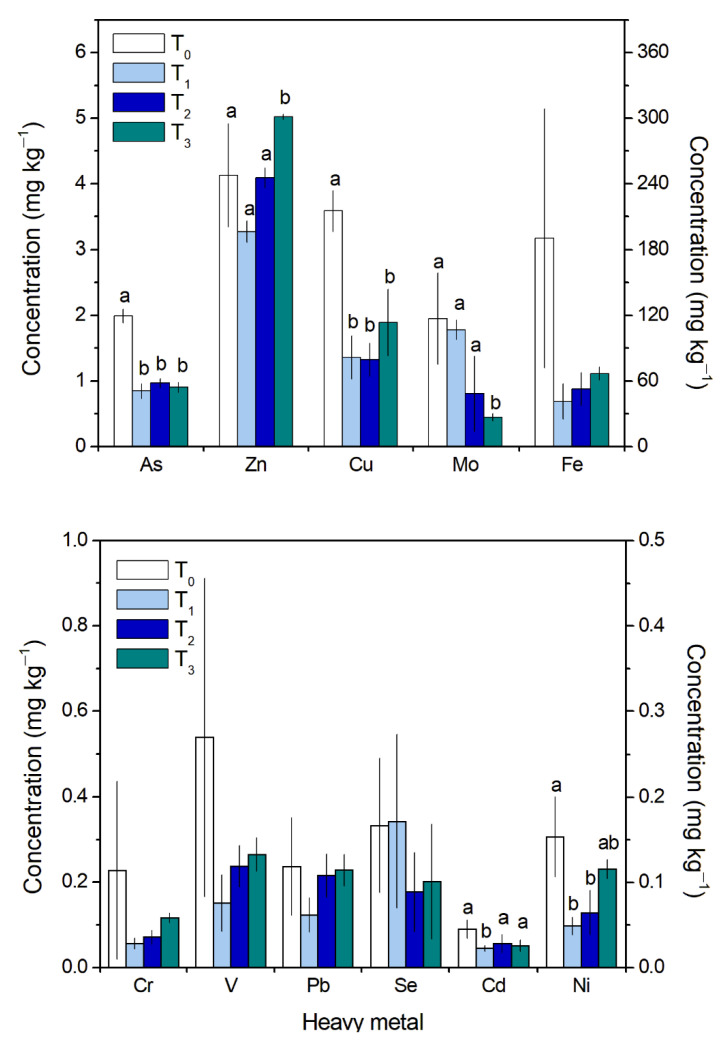
Heavy metal concentrations (mg kg^–1^ fresh tissue) in *Gracilaria chilensis* biomass from Los Albatros Beach (T_0_) and before seawater cleaning and tap water submersion (T_1_). T_2_ and T_3_ correspond to 14 and 21 days of culture after the first submersion, respectively. For As, Zn, Cu, Mo, Cr, V, Pb, and Se, the concentration is indicated on left Y-axis. For Fe, Cd, and Ni, the concentration is indicated on the right Y-axis. Different letters indicate statistically different concentrations for each metal along the experimental setup. The data shown are the means ± SD, *n* = 3.

The concentrations of most metals in *G. chilensis* biomass after the first tap water submersion were lower than those measured in the naturally occurring biomass (T_0_, Figure 1). For As and Cu, the concentrations remained without any significant variation throughout the culture after the first tap water submersion, but at 50% and 62% below the basal concentration (T_0_; Figure 1), respectively. The highest concentrations for As and Cu were 2.1 and 3.8 ± 0.3 mg kg^−1^, respectively, and the lowest were 0.8 and 1.1 ± 0.1 mg kg^−1^, respectively. The Mo concentration decreased throughout the experiment after the first submersion, reaching the lowest concentrations at 21 days (0.44 mg kg^−1^) (Figure 1). The Zn and Ni concentrations decreased after the first tap water submersion but then increased during the culture. Zn concentrations in particular reached values 22% higher than those of the naturally occurring biomass (5.02 ± 0.03 mg kg^−1^ vs. 4.13 ± 0.79 mg kg^−1^) (Figure 1). The Cd concentrations showed significant differences only after the tap water submersion (Figure 1). At 14 and 21 days, the Cd concentrations did not evidence any significant differences and remained between 0.02 mg kg^−1^ and 0.03 mg kg^−1^. The Fe, Cr, V, Pb, and Se concentrations did not show any significant differences, but they showed a trend of reduced concentrations at 14 days of culture in relation to the naturally occurring *G. chilensis* biomass (except for Pb) (Figure 1). Fe was the metal with the highest concentration value in the algal tissue at T_0_ (124–327 mg kg^−1^), 97 to 4000 times higher than those obtained for the other metals (Figure 1).

### 2.2. Percentage of Depuration of Heavy Metals

From the measured concentrations of the heavy metals in the *G. chilensis* biomass, the percentages of depuration (PD) were calculated (Equation (1) in the Method Section) during the entire experimental period, taking the initial concentration for each metal as the basal value (T_0_).

The obtained values show a clear trend of depuration of the heavy metals in the culture after the first tap water submersion (Table 1). The PD was higher in the first week of culture and maintained a positive value for the rest of the experimental time. Contrarily, Zn showed a clear bioaccumulation in the biomass during the second and third weeks of culture.

### 2.3. Normative Comparison

At 21 days of culture after the first tap water submersion, most of the heavy metal concentrations of the biomass reached lower values than some regulatory limits for human consumption (Table 2). For example, the Pb concentration was below the regulatory limits for the Canadian regulation, Codex Alimentarius, and Decree 997, which approves food health regulations in Chile. The Cd concentrations remained between the minimum and maximum limits for Decree 997. The As concentration exceeded the regulatory limits established by Codex Alimentarius and Decree 997. In the case of Fe, its concentrations exceeded only the regulatory limits of Decree 997. All other metal concentrations were lower than these regulatory limits, as shown in Table 2. It is important to mention that they are not indicated in any normative “seaweed products”.

### 2.4. Daily Growth Rates of Gracilaria chilensis Biomass

**Figure 2:**
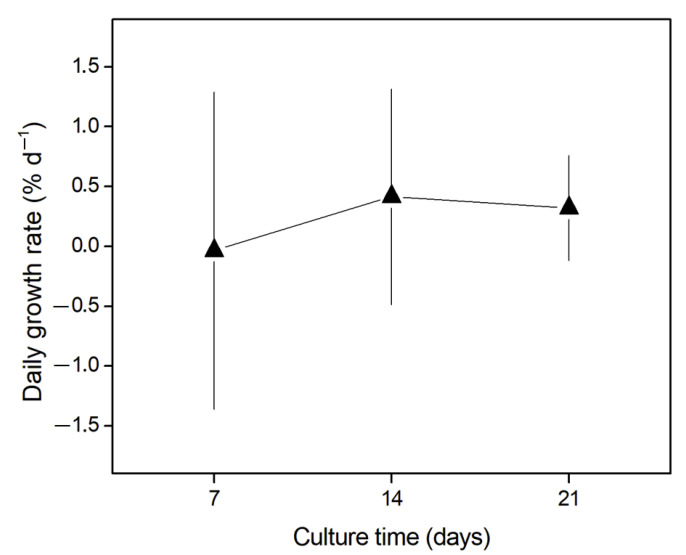
Daily growth rate (DGR, % d^−1^) of *Gracilaria chilensis* biomass during 21 days of culture. Error bars indicate standard deviation. There were no significant differences between the averages according to the analysis of variance (*p* > 0.05).

The daily growth rate (DGR, % d^−1^) results are presented in Figure 2. The DGR of *G. chilensis* did not increase over time (days of culture, F_(2, 0.339)_ = 0.185; *p* > 0.05). Table 3 shows the statistical results of the comparisons of mean DGR of the different culture times. During the culture time, the highest DGR was recorded after 14 days of culture (DGR = 0.41 ± 0.89% d^−1^), and the lowest DGR occurred after the first week of culture (DGR = −0.03 ± 1.32% d^−1^). Overall, the experimental setup did not negatively impact the daily growth of the biomass. It is important to mention that no variability was determined for the temperature, photosynthetically active radiation (PAR), salinity, and dissolved oxygen over the entire experimental period (Supplementary Material Table S2).

## 3. Discussion

We designed a simple operational heavy metal depuration protocol from *Gracilaria chilensis* biomass, utilizing 100 µm-filtered seawater and tap water remotion. This protocol permitted the reduction of 10 heavy metals, including As, Cu, Pb, and Cd at 7 days of culture. The percentage of depuration ranged from 32 to 92% at 7 days, which was maintained for up to 21 days of culture, except for Zn. Throughout the duration of the experiment, the monitored physicochemical parameters (temperature, salinity, and dissolved oxygen, among others) were stable (Supplementary Material Table S2). Thus, these results allowed us to deduce that the main effect on the depuration was produced by the water changes during the culture but not by any other physicochemical variable. Consequently, the experimental setup was successful for the depuration of the majority of the analyzed heavy metals, highlighting the importance of outdoor cultures as tools of sustainability.

The high concentrations of several heavy metals in *G. chilensis* before the experimental setup indicate a substantial bioconcentration of these heavy metals in the biomass from the impact site (Los Albatros Beach) (Figure 1). In fact, similar results for bioconcentrations have been reported for diverse seaweeds [38,39,40], finding that red algae have a great capacity to incorporate metals from seawater. For example, Wang et al. (2013) [39] concluded that *Gracilaria lemaneiformis* exposed to seawater enriched with Pb, Cu, and Cd bioaccumulated these metals up to 30.1, 3.75, and 10.15 times the control condition, respectively. Recently, Luo et al. (2021) [40] indicated the importance of *G. lemaneiformis* as a remover of Zn, Cu, Pb, and Cd, influencing heavy metal cycling in the environment. The mechanisms associated with the bioaccumulation of these metals are primarily superficial adsorption followed by a slower, metabolically arranged intracellular uptake [31,32]. In fact, Cd bioaccumulation in *Gracilaria tenuistipitata* has been suggested to occur through biphasic kinetics, where an electrostatic interaction occurs first in a rapid manner followed by a slower process that occurs after cell wall saturation, mediated by metabolic processes contributing to absorption [41]. It is important to mention that, depending on the species, the heavy metal accumulation order depends on the affinity to a certain metal by the organism [42]. The order of the bioconcentration of heavy metals in this study was similar to that registered in *G. lameneiformis* (Zn > Cu > Pb > Cd) [40].

The basal concentration of the heavy metals determined in *G. chilensis* is indicative of highly polluted marine zones. In fact, similar results have been determined in the kelp *Macrocystis pyrifera* [29] and in *Lessonia spicata* [43] from the same zone of study. Recently, it was determined that a short pulse of a high concentration of heavy metal in the early stages of *M. pyrifera* growth negatively affects its development and morphometry as well as its role as an ecosystem engineer due to a negative alteration in the species composition [44]. In addition, a strong trophic transference of the heavy metals from seaweed biomass to the sea urchin *Tetrapygus niger* was determined since the concentrations measured in the latter were higher than in the seaweed biomass [29]. This consumption negatively affects the life cycle of the sea urchin [28]. All these results show the impacts of heavy metals on marine communities and highlight the necessity of incorporating significant actions to reduce anthropogenic emissions to the environment.

The heavy metal concentration analysis (Figure 1) showed that there were significant reductions in As, Cu, Mo, Cd, and Ni during the treatment of the biomass and culture but not in Fe, V, Pb, and Se. In red algae, some authors have proposed that the bioaccumulation of heavy metals is proportional to the exposure time and their concentrations in the water [39,45]. Thus, the culture of *G. chilensis* from impacted sites should be at least 4 weeks long to ensure major reductions in all heavy metals considered in the study and to reduce those that exceeded the permitted values of standard limits (As and Fe).

In this work, Zn was the only element studied that showed a greater concentration in the Quintay seawater than at the impacted site (Los Albatros Beach, Supplementary Material Figure S1). We suggest that this bioaccumulation is due to the high Zn concentration in Quintay seawater, which was evidenced in the biomass at the end of the experimental period. Likewise, it is important to mention that metal accumulation depends on several factors, including the nature of the element and its affinity with diverse chemical groups [46]. The cell walls of *G. chilensis* and other species are composed of cellulose, alginate (polysaccharides), and lipids, while proteins provide amino acids, phosphate, and hydroxyl, thiol-rich, and carboxyl functional groups, which all possess high affinities for binding metal ions [47], particularly Zn [47,48]. For example, in *Gracilaria corticata*, the pseudo-first-order kinetic model suggested that zinc biosorption is based on a chemical reaction involving an electron exchange between the alga and the metal [49], consequently influencing the high bioaccumulation of this element in *Gracilaria* biomass.

The concentrations of Fe in *G. chilensis* were similar to those registered in *G. corticata* and *Gracilaria edulis,* where the Fe content, with respect to other metals (i.e., Cu, Mg, and Zn), was 20 to 97 times higher [50]. Fe is an essential element in the metabolism of algae, and it can show temporal variability [51]. Fe is also a good predictor of the presence of other metals such as Mn, Cu, Zn, Ni, and V [46], and its content has been shown to be high in different species of algae (*Sargassum* sp., 1569 mg kg^–1^ dw; *Ulva* sp., 575 mg kg^–1^ dw; 311 mg kg^–1^ dw; *Porphyra* sp., 155 mg kg^–1^ dw, and *Gracilariopsis* sp., 1959 mg kg^–1^ dw) [52]. It is important to mention that the results obtained in this study are consistent with those of other studies that demonstrated a relation between the metal concentration in the seaweed biomass, e.g., *M. pyrifera* [53,54], *Ulva lactuca,* and *Gracilaria blodgettii* [55], and those present in the water column or culture medium.

The depuration of heavy metals from *G. chilensis* biomass was favorable, as was their maintenance in the culture. In this case, and considering the DGR, a positive trend can be observed (Figure 2). Although the DGR values were not significantly different, these results are important because they show that the culture under the experimental conditions did not show a loss of biomass and thus can help in establishing an acclimatization protocol for larger volumes of biomass without the need to enrich the seawater with an external source of nutrients [56,57]. Moreover, the developed protocol represents a starting point for new studies aiming to increase the biomass in outdoor cultures and at the same time increase the depuration of heavy metals in algal biomass from contaminated environments without generating losses in the treated biomass.

## 4. Conclusions

Due to the importance of seaweeds for human consumption, and in pharmaceutical and/or nutraceutical application, the use of cultivation systems is necessary to avoid harvesting from natural populations and overexploitation of the resource. Several seaweed species have great potential in terms of productivity and are the source of several human commodities, but the presence of heavy metals could alter the crops and lead to potential risks for consumption. Thus, a culture with a depuration process prior to the consumption or extraction of the compounds of interest is necessary to reduce the risk to health and to avoid the final products containing these contaminants in their final composition.

## 5. Materials and Methods

### 5.1. Seaweed Collection

Along the coast of Chile, there are several industrial parks; one of them is the industrial area of Quintero–Puchuncaví, located on the central coast of Chile (32°45′ S, 71°29′ W). This industrial park began operation in 1961, and it is characterized by high pollution levels due to historical discharges of petroleum, gaseous pollutants, and atmospheric particulates as well as the disposal of heavy metals from various industrial facilities [43,58,59]. During spring of 2021, we collected 15 kg of *G. chilensis* from a beach of this zone, namely Los Albatros Beach (32°46′57.21″ S, 71°31′27.52″ W), which is characterized by high levels of heavy metals in the seawater (Supplementary Material Figure S1). After the collection, the biomass was stored in plastic bags and transported in coolers with ice packs to the Centro de Investigación Marina de Quintay (CIMARQ) located in Valparaíso (33°11′35.76″ S, 71°42′08.43″ W). Subsequently, the biomass was manually cleaned by removing all visible organisms and organic material that were present. The treated biomass was used for the setup of the following experiments. All the materials utilized for the collection and transport were previously prepared according to [43].

### 5.2. Experimental Setup, Open-System Culture Conditions, and Measured Parameters 

For the initial determination of heavy metal concentrations (basal condition), approximately 350 g of fresh biomass of *G. chilensis* was taken in triplicate after the manual cleaning (T_0_ condition). Then, another 350 g (in triplicate) was submerged in tap water (25 L) for 1 h (T_1_ condition). In both cases, the biomass was stored at −80 °C before the heavy metal analyses by inductively coupled plasma mass spectrometry (ICP–MS) according to method 6020B (USEPA method). Briefly, 1 g of dry grounded sample was digested with 10 mL of a 7:2:1 mixture of HNO_3_ + HCl + H_2_O_2_ (67, 37, and 30% *w*/*v*, respectively). After 10 min of gently stirring, samples were transferred to special vessels, and the digestion process was performed in a Titan MPS digester (N3130100 model, Perkin Elmer, UK). The digestion program used was as follows: 5 min to 250 W, 5 min to 350 W, 10 min to 500 W, and 5 min to ventilation. Finally, the samples were transferred to a 100 mL volumetric flask with 2% *v*/*v* HNO_3_ and filtered with 0.22 µm syringe filter for ICP–MS analysis. For each series of analyses, reagent blanks and a calibration curve from 0.01–100 ng/mL were prepared (R^2^ > 0.999) (ICP multi-element standard solution XVI, Merck, Germany). The limits of detection for the ICP–MS measurements of each element were as follows: As and Cu = 0.025 mg kg^−1^; Cd = 0.005 mg kg^−1^; Zn = 0.250 mg kg^−1^; Cr, Mo, Ni and V = 0.013 mg kg^−1^; Pb = 0.002 mg kg^−1^; and Fe and Se = 0.125 mg kg^−1^.

Between 6 and 8 kg of tap-water-cleaned biomass was placed in three independent 400 L culture tanks with 300 L of 100 μm-filtered seawater, a constant flow rate of 11.35 L/min, and aeration. The tanks were covered with 80% Raschel shade mesh to avoid direct light radiation, reaching between 80–150 µmol m^−2^ s^−1^. The tanks and the biomass were cleaned weekly using tap water, and the cultivation period lasted 21 days.

At 14 and 21 days of culture (the T_2_ and T_3_ conditions, respectively), about 350 g of algal tissue (in triplicate) was collected for heavy metal measurements after a 1 h submersion in tap water, as conducted at T_1_.

During the entire experiment, various physicochemical parameters were determined in seawater, such as the temperature (DT8750E infrared thermometer, RoHS), photosynthetically active radiation (PAR) light intensity (Quantum MQ-510-m, Apogee), pH, oxidoreduction potential (ORP), salinity, and dissolved oxygen (Multiparameter Meter HI9829, Hanna Instruments).

### 5.3. Percentage of Depuration (PD) of Heavy Metals

To determine the variations in the concentration of heavy metals in *G. chilensis* biomass, the PD was calculated according to the following formula:

$$PD\;\left(\%\right)=\frac{C_{0}-C_{t}}{C_{0}}\times 100\%$$

where *C*_t_ is the heavy metal concentration in the T_1_, T_2_, and T_3_ treatments and *C*_0_ is the basal concentration.

### 5.4. Daily Growth Rate (DGR)

To estimate the increase in algal biomass with respect to time during the whole culture period, the DGR (% d^−1^) was determined at 7, 14, and 21 days of culture by the following equation:

$$DGR\;\left(\%\;d^{-1}\right)=\left[{\left(\frac{w_{t}}{w_{t-1}}\right)}^{\frac{1}{t}}-1\right]100\%$$

where *w_t_* is the mass after seven days of culture; *w_t−1_* is the mass during the previous week; and *t* is the time expressed in days [60].

### 5.5. Data Analyses

Quantitative analyses were performed to determine the effect of the treatments on heavy metal concentrations in *G. chilensis* biomass. To evaluate significant interactions between the heavy metal concentration, the percentage of depuration, and DGR vs. culture time, a one-way ANOVA was performed. Tukey’s tests were calculated to determine in which conditions such differences exist. To evaluate the assumptions of normality and the homogeneity of variances, the Shapiro–Wilk and Levene’s tests were applied. All statistical analyses were performed in the R package (R Development Core Team 2022) statistical environment, and significances were set at *p* < 0.05. The heavy metal concentrations after 21 days of culture were compared with the following national and international regulations for acceptable limits in food for human consumption (mg kg^−1^, fresh tissue): the maximum metal levels according to Canadian guidelines for chemical contaminants and toxins in fish and fish products; the maximum metal level according to Commission regulation (EC) No. 1881/2006, European Union; the maximum metal levels according to CXS193e (Codex Alimentarius); and Decree 997 that approves food health regulations in Chile.

## Figures and Tables

**Table 1:** Ranges of percentage of depuration (PD) of heavy metals in *Gracilaria chilensis* biomass under the proposed methodology. T_1_ represents the values calculated after tap water submersion. T_2_ and T_3_ correspond to 14 and 21 days of culture after the first submersion, respectively. Positive values mean a decrease in the concentration, and negative values indicate a bioaccumulation of the heavy metals. For statistical results see Supplementary Material Table S1.

Ranges of Percentage of Depuration						
	T_1_		T_2_		T_3_	
	Min	Max	Min	Max	Min	Max
As	54	60	49	55	53	57
Zn	−5	32	−25	18	−4	−55
Cu	54	67	58	69	36	56
Cr	−38	90	−29	87	−106	73
Mo	−45	39	20	94	69	81
Pb	−32	75	−116	48	−158	33
Se	−84	39	−38	76	30	56
V	8	92	1	78	−43	74
Fe	54	92	42	87	41	81
Cd	31	58	13	50	37	51
Ni	65	71	34	72	−6	40

**Table 2:** Comparison of heavy metal concentrations measured in *G. chilensis* at 21 days of culture and regulatory limits (mg kg^−1^) of heavy metals in the tissue of seafood products for human consumption.

Metal	Concentration at 21 Days (mg kg^−1^) *	Maximum Metal Levels According to Canadian Guidelines for Chemical Contaminants and Toxins in Fish and Fish Products	Maximum Metal Levels According to Commission Regulation (EC) No. 1881/2006, European Union	Maximum Metal Levels According to CXS_193e (Codex Alimentarius)	Decree 997 Approves Food Health Regulations (Chile)
As	0.90 ± 0.07	3.5 ^a^	n.i.	0.1–0.5 ^c^	0.01–0.5 ^e^
Zn	5.02 ± 0.03	n.i.	n.i.	n.i.	100.0 ^f^
Cu	1.9 ± 0.5	n.i.	n.i.	n.i.	10.0 ^f^
Cr	0.12 ± 0.01	n.i.	n.i.	n.i.	n.i.
Mo	0.45 ± 0.05	n.i.	n.i.	n.i.	n.i.
Pb	0.23 ± 0.04	0.5 ^a^	0.3–1.5 ^b^	0.3 ^d^	2.0 ^g^
Se	0.20 ± 0.01	n.i.	n.i.	n.i.	0.30 ^h^
V	0.27 ± 0.04	n.i.	n.i.	n.i.	n.i.
Fe	66.8 ± 5.7	n.i.	n.i.	n.i.	0.2–50 ^e^
Cd	± 0.01	n.i.	0.05–1.0 ^b^	2.0 ^d^	0.01–0.5 ^i^
Ni	0.12 ± 0.01	n.i.	n.i.	n.i.	n.i.

^a^ In fish protein concentrate. ^b^ In muscle meat of fish and/or bivalve mollusks. ^c^ In all categories shown except for natural mineral water. ^d^ In marine bivalve mollusks. ^e^ Minimum and maximum values of all products shown in the list of the standard. ^f^ Other products from those listed in the standard. ^g^ Canned, fresh, chilled, and frozen seafood. ^h^ In solid products. ^i^ Edible table salt mineral water. n.i. indicates the absence of regulatory limits for that metal. * Average ± SD fresh biomass.

**Table 3:** Results of one-way ANOVA for culture time (days) and daily growth rate (DGR, % d^−1^) of *Gracilaria chilensis* biomass under the proposed methodology.

	DF	Sum Sq	Mean Sq	F	*p* Value
Time (days of culture)	2	0.339	0.1694	0.185	0.84
Residuals	6	5.504	0.9174		

## Data Availability

Not applicable.

## Supplementary Materials

The following supporting information can be downloaded at: https://www.mdpi.com/article/10.3390/molecules27206832/s1, Table S1: Results of one-way ANOVA for percentage of depuration of heavy metals in *G. chilensis* biomass; Table S2: Physicochemical parameters measured in the seawater during the experimental culture. T1, T2, and T3 correspond to 7, 14, and 21 days of culture after the first biomass submersion, respectively; Figure S1: Concentrations of some heavy metals studied in the seawater from Los Albatros Beach and Quintay. Bars represent means ± SD (*n*= 3). Identical letters indicate no significant difference (*p* > 0.05).

## Sample Availability

Not available.

## References

[1] Anis M., Ahmed S., Hasan M.M. (2017). Algae as nutrition, medicine and cosmetic: The forgotten history, present status and future trends. World J. Pharm. Pharm. Sci..

[2] Ganesan A.R., Tiwari U., Rajauria G. (2019). Seaweed nutraceuticals and their therapeutic role in disease prevention. Food Sci. Hum. Wellness.

[3] Rimmer M.A., Larson S., Lapong I., Purnomo A.H., Pong-Masak P.R., Swanepoel L., Paul N.A. (2021). Seaweed Aquaculture in Indonesia Contributes to Social and Economic Aspects of Livelihoods and Community Wellbeing. Sustainability..

[4] FAO (2022). The State of World Fisheries and Aquaculture; Towards Blue Transformation.

[5] SERNAPESCA (2021). Anuario Estadístico de Pesca y Acuicultura. Servicio Nacional de Pesca, Valparaíso. http://www.sernapesca.cl/informacion-utilidad/anuarios-estadisticos-de-pesca-y-acuicultura..

[6] Arakaki N., Schmidt W.E., Carbajal P., Fredericq S. (2015). First occurrence of *Gracilaria chilensis*, and distribution of *Gracilariopsis lemaneiformis* (Gracilariaceae, Gracilariales) in Peru on the basis of *rbc*L sequence analysis. Phytotaxa..

[7] Brito N. (2019). El Oro Negro de Pitipalena. Rescate Patrimonial de la Extracción del Pelillo (Gracilaria chilensis) en Puerto Raúl Marín Balmaceda.

[8] Dillehay T.D., Ramírez C., Collins M.B., Rossen J., Pino-Navarro J.D. (2008). Monte Verde: Seaweed, food, medicine, and the peopling of South America. Science.

[9] Torres P., Santos J.P., Chow F., dos Santos D.Y.A.C. (2019). A comprehensive review of traditional uses, bioactivity potential, and chemical diversity of the genus *Gracilaria* (Gracilariales, Rhodophyta). Algal Res..

[10] Le Y., Wang B., Xue M. (2022). Nutraceuticals use and type 2 diabetes mellitus. Curr. Opin. Pharmacol..

[11] Rajasekaran A., Chackalamannil S., Rotella D., Ward S.E. (2017). 1.05—Nutraceuticals. Comprehensive Medicinal Chemistry III.

[12] Hafting J.T., Craigie J.S., Stengel D.B., Loureiro R.R., Buschmann A.H., Yarish C., Edwards M.D., Critchley A.T. (2015). Prospects and challenges for industrial production of seaweed bioactives. J. Phycol..

[13] Pinto C., Ibáñez M.R., Loyola G., León L., Salvatore Y., González C., Barraza V., Castañeda F., Aldunate R., Contreras-Porcia L. (2021). Characterization of an *Agarophyton chilense* Oleoresin Containing PPARgamma Natural Ligands with Insulin-Sensitizing Effects in a C57Bl/6J Mouse Model of Diet-Induced Obesity and Antioxidant Activity in *Caenorhabditis elegans*. Nutrients.

[14] Guillemin M.-L., Faugeron S., Destombe C., Viard F., Correa J.A., Valero M. (2008). Genetic Variation in Wild and Cultivated Population of the Haploid-diploid red Alga *Gracilaria chilensis*: How farming practices favor asexual reproduction and heterozygosity. Evolution.

[15] Westermeier R., Rivera P.J., Gomez I. (1988). El uso de mangas de polietileno como sustrato en el repoblamiento de *Gracilaria* sp. (Rhodophyta, Gigartinales) en el sur de Chile. Gayana.

[16] Avila M., Aroca G., Rodríguez D., Riquelme R., Piel M.I., Ramírez M.E., De Zarate C. (2019). Manual de Buenas Prácticas para el Cultivo de Pelillo (Agarophyton chilensis ex Gracilaria chilensis).

[17] Saavedra S., Henríquez L., Leal P., Galleguillos F., Cook S., Cárcamo F. (2019). Cultivo de Macroalgas: Diversificación de la Acuicultura de Pequeña Escala en Chile.

[18] Campbell I., Macleod A., Sahlmann C., Neves L., Funderud J., Øverland M., Hughes A.D., Stanley M. (2019). The Environmental Risks Associated with the Development of Seaweed Farming in Europe—Prioritizing Key Knowledge Gaps. Front. Mar. Sci..

[19] Walkinshaw C., Lindeque P.K., Thompson R., Tolhurst T., Cole M. (2020). Microplastics and seafood: Lower trophic organisms at highest risk of contamination. Ecotoxicol. Environ. Saf..

[20] Yokoya N., Oliveira E.C. (1992). Temperature responses of economically important red algae and their potential for mariculture in Brazilian waters. J. Appl. Phycol..

[21] Yokoya N., Oliveira E.C. (1992). Effects of salinity on the growth rate, morphology and water content of some Brazilian red algae of economic importance. Cienc. Mar..

[22] Rivas J., Núñez A., Piña F., Erazo F., Castañeda F., Araya M., Meynard A., Contreras-Porcia L. (2021). Indoor culture scaling of *Gracilaria chilensis* (Florideophyceae, Rhodophyta): The effects of nutrients by means of different culture media. Revista de Biología Marina y Oceanografía.

[23] Titlyanov E.A., Titlyanova T.V. (2010). Seaweed cultivation: Methods and problems. Russ. J. Mar. Biol..

[24] Rees A. (2003). Safety factors and nutrient uptake by seaweeds. Mar. Ecol. Prog. Ser..

[25] Harrison P., Hurd C. (2001). Nutrient physiology of seaweeds: Application of concepts to aquaculture. Cah. Biol. Mar..

[26] Reef R., Pandolfi J.M., Lovelock C. (2012). The effect of nutrient enrichment on the growth, nucleid acid concentrations, and elemental stoichiometry of coral reef macroalgae. Ecol. Evol..

[27] Ali H., Khan E. (2018). Bioaccumulation of non-essential hazardous heavy metals and metalloids in freshwater fish. Risk to human health. Environ. Chem. Lett..

[28] Latorre-Padilla N., Meynard A., Oyarzun F.X., Contreras-Porcia L. (2021). Ingestion of contaminated kelps by the herbivore *Tetraphygus niger*: Negative effects on food intake, growth, fertility, and early development. Mar. Pollut. Bull..

[29] Latorre-Padilla N., Meynard A., Rivas J., Contreras-Porcia L. (2021). Transfer of Pollutants from *Macrocystis pyrifera* to *Tetraphygus niger* in a Highly Impacted Coastal Zone of Chile. Toxics.

[30] Peng Z., Guo Z., Wang Z., Zhang R., Wu Q., Gao H., Wang Y., She Z., Lek S., Xiao J. (2022). Species-specific bioaccumulation and health risk assessment of heavy metal in seaweeds in tropic coasts of South China Sea. Sci. Total Environ..

[31] Garnham G.W., Codd G.A., Gadd G.M. (1992). Accumulation of Cobalt, Zinc and Manganese by the Estuarine Green Microalga *Chlorella salina* Inmobilized in Alginate Microbreads. Environ. Sci. Technol..

[32] Andrade S., Contreras L., Moffett J.W., Correa J.A. (2006). Kinetics of copper accumulation in *Lessonia nigrescens* (Phaeophyceae) under conditions of environmental oxidative stress. Aquat. Toxicol..

[33] Contreras L., Moenne A., Correa J.A. (2005). Antioxidant responses in *Scytosiphon lomentaria* (Phaeophyceae) Inhabiting Cooper-Enriched coastal environments. J. Phycol..

[34] Sordet C., Contreras-Porcia L., Lovazzano C., Goulitquer S., Andrade S., Potin P., Correa J.A. (2014). Physiological plasticity of *Dictyota kunthii* (Phaeophyceae) to Cooper excess. Aquat. Toxicol..

[35] Contreras-Porcia L., Meynard A., López-Cristoffanini C., Latorre N., Kumar M. (2017). Marine Metal Pollution and Effects on Seaweed Species. Syst. Biol. Mar. Ecosyst..

[36] Mohamed S.F., Borik R.M. (2013). Modern Trends in Using Marine Algae for Treatment of Aquatic Pollution. Int. J. ChemTech Res..

[37] Araya M., Rivas J., Sepúlveda G., Espinoza-González C., Lira S., Meynard A., Blanco E., Escalona N., Ginocchio R., Garrido-Ramírez E. (2021). Effect of Pyrolysis Temperature on Copper Aqueous Removal Capability of Biochar Derived from the Kelp *Macrocystis pyrifera*. Appl. Sci..

[38] Sudharsan S., Seedevi P., Ramasamy P., Subhapradha N., Vairamani S., Shanmugam A. (2012). Heavy metal accumulation in seaweeds and sea grasses along southeast coast of India. J. Chem. Pharm. Res..

[39] Wang Z., Wang X., Ke C. (2013). Bioaccumulation of trace metals by the live macroalga *Gracilaria lemaneiformis*. J. Appl. Phycol..

[40] Luo H., Wang Q., Zhang C., Zhang L., Yang Y. (2021). Bioaccumulation and release of heavy metals during growth and decomposition of cultivated *Gracilaria lemaneiformis*. Mar. Pollut. Bull..

[41] Hu S., Tang C.H., Wu M. (1996). Cadmium accumulation by several seaweeds. Sci. Total Environ..

[42] Sun X., Liu Z., Jiang Q., Yang Y. (2019). Concentrations of various elements in seaweed and seawater from Shen’ao bay, Nan’ao Island, Guangdong coast, China: Environmental monitoring and the bioremediation potential of the seaweed. Sci. Total Environ..

[43] Oyarzo-Miranda C., Latorre N., Meynard A., Rivas J., Bulboa C., Contreras-Porcia L. (2020). Coastal pollution from the industrial park Quintero Bay of central Chile: Effects on abundance, morphology, and development of the kelp *Lessonia spicata* (Phaeophyceae). PLoS ONE.

[44] Jara-Yáñez R., Meynard A., Acosta G., Latorre-Padilla N., Oyarzo-Miranda C., Castañeda F., Piña F., Rivas J., Bulboa C., Contreras-Porcia L. (2021). Negative consequences on the Growth, Morphometry and Community Structure of the Kelp *Macrocystis pyrifera* (Phaeophyceae, Ochrophyta) by a Short Pollution Pulse of Heavy Metals and PAHs. Toxics.

[45] Ali A.Y.A., Idris M.A., Eltayeb M.A.H., El-Zahhar A.A., Ashraf I.M. (2021). Bioaccumulation and health risk assessment of toxic metals in red algae in Sudanese Red Sea coast. Toxin Rev..

[46] Benabdallah N., Harrache D., Mir A., de la Guardia M., Benhachem F.Z. (2017). Bioaccumulation of trace metals by red alga *Corallina elongata* in the coast of Beni Saf, west coast, Algeria. Chem. Int..

[47] Foday E.H., Bo B., Xu X. (2021). Removal of Toxic Heavy Metals from Contaminated Aqueous Solutions Using Seaweeds: A Review. Sustainability.

[48] Stengel D.B., Macken A., Morrison L., Morley N. (2004). Zinc concentrations in marine macroalgae and a lichen from western Ireland in relation to phylogenetic grouping, habitat and morphology. Mar. Pollut. Bull..

[49] Anilkumar B., Babu N., Kavitha G. (2016). Biosorption of Zinc on to *Gracilaria corticata* (Red Algae) Powder and Optimization using Central Composite Design. J. Appl. Sci. Eng. Methodol..

[50] Rosemary T., Arulkumar A., Paramasivam S., Mondragon-Portocarrero A., Miranda J.M. (2019). Biochemical, Micronutrient and Physicochemical Properties of the Dried Red Seaweeds *Gracilaroa edulis* and *Gracilaria corticata*. Molecules.

[51] Jayasankar R., Paliwal K. (2002). Seasonal variation in the elemental composition of *Gracilaria* species of the Gulf of Mannar, Tamil Nadu coast. Seaweed Res. Util..

[52] García-Casal M.N., Pereira A.C., Leets I., Ramírez J., Quiroga M.F. (2007). High Iron Content and Bioavailability in Humans from Four Species of Marine Algae. J. Nutr..

[53] Evans L.K., Edwards M.S. (2011). Bioaccumulation of cooper and zinc by the giant kelp *Macrocystis pyrifera*. Algae.

[54] Lares M.L., Flores-Muñoz G., Lara-Lara R. (2002). Temporal variability of bioavaliable Cd, Hg, Zn, Mn and Al in an upwelling regime. Environ. Pollut..

[55] Wang W.-X., Dei R.C.H. (1999). Kinetic measurements of metal accumulation in two marine macroalgae. Mar. Biol..

[56] Capo T.R., Jaramillo J.C., Boyd A.E., Lapointe B.E., Serafy J.E. (1999). Sustained high yields of *Gracilaria* (Rhodophyta) grown in intensive large-scale culture. J. Appl. Phycol..

[57] Yu J., Yang J.-F. (2008). Physiological and biochemical response of seaweed *Gracilaria lemaneiformis* to concentration changes of N and P. J. Exp. Mar. Biol. Ecol..

[58] Larsson N. (2020). The brutal reality of life inside one of the world’s most polluted cities. Wired Magazine.

[59] Gayo E.M., Muñoz A.A., Maldonado A., Lavergne C., Francois J.P., Rodríguez D., Klock-Barría K., Sheppard P.R., Aguilera-Betti I., Alonso-Hernández C. (2022). A Cross-Cutting Approach for Relating Anthropocene, Environmental Injustice and Sacrifice Zones. Earth’s Future.

[60] Yong Y.S., Yong W.T.L., Anton A. (2013). Analysis of formulae for determination of seaweed growth rate. J. Appl. Phycol..

