# Supplementary material for: Heavy Metal Depuration Steps for Gracilaria chilensis in Outdoor Culture Systems
Source: Molecules. 2022 Oct 12;27(20):6832. doi: 10.3390/molecules27206832 (PMC9611025; doi:10.3390/molecules27206832)
Supplement: Supplementary file 1 [file molecules-27-06832-s001.zip › molecules-1865901-supplementary.pdf]

molecules

Supplementary Material

Heavy metal depuration steps for *Gracilaria chilensis* in outdoor culture systems

Jorge Rivas [1,2,3,4], Florentina Piña[1,2,3,4,5], Matías Araya[1,2,3,4], Nicolás Latorre-Padilla[1,2,3,4], Benjamín Pinilla-Rojas[1,2,3,4], Sofía Caroca[1,2,3,4], Francisca C. Bronfman[6] and Loretto Contreras-Porcia[1,2,3,4,*]

1 Departamento de Ecología y Biodiversidad, Facultad de Ciencias de la Vida, Universidad Andres Bello, Santiago 8370251, Chile
2 Centro de Investigación Marina Quintay (CIMARQ), Facultad de Ciencias de la Vida, Universidad Andres Bello, Quintay, Valparaíso, 2531015, Chile
3 Center of Applied Ecology and Sustainability (CAPES), Santiago 8331150, Chile
4 Instituto Milenio en Socio-Ecología Costera (SECOS), Santiago 8370251, Chile
5 Programa de Doctorado en Biotecnología, Facultad de Ciencias de la Vida, Universidad Andres Bello, Santiago 8370251, Chile
6 Institute of Biomedical Science (ICB), Faculty of Medicine, Universidad Andres Bello, Echaurren 183, Santiago 8320000, Chile
* Correspondence: lorettocontreras@unab.cl

Table S1. Results of one-way ANOVA for percentage of depuration of heavy metals in *G. chilensis* biomass.

		DF	Sum Sq	Mean Sq	F	P value
As	Time (days of culture)	2	56.6731	28.3365	3.229	0.11
	Residuals	6	52.6582			
Zn	Time (days of culture)	2	2806.81	1403.4	2.61	0.15
	Residuals	6	3226.69			
Cu	Time (days of culture)	2	447.25	223.625	3.514	0.09
	Residuals	6	381.823			
Cr	Time (days of culture)	2	3084.94	1542.47	0.2705	0.77
	Residuals	6	34217.4			
Mo	Time (days of culture)	2	9092.91	4546.46	4.034	0.07
	Residuals	6	6762.39			
Pb	Time (days of culture)	2	6386.85	3193.42	0.4246	0.67
	Residuals	6	45120.9			
Se	Time (days of culture)	2	3898.41	1949.2	0.6917	0.53
	Residuals	6	16908.8			
V	Time (days of culture)	2	1152.87	576.436	0.2434	0.79
	Residuals	6	14211.3			
Fe	Time (days of culture)	2	312.167	156.084	0.3476	0.71
	Residuals	6	2693.83			
Cd	Time (days of culture)	2	178.03	89.015	0.384	0.68
	Residuals	6	1390.76			
Ni	Time (days of culture)	2	3651.44	1825.72	5.644	0.04
	Residuals	6	1940.89			

Table S2. Physico–chemical parameters measured in the seawater during the experimental culture. T1, T2, and T3 correspond to 7, 14, and 21 days of culture after the first biomass submersion, respectively.

	pH	ORP (mV)	DO (ppm)	Salinity (PSU)	Turbidity (FNU)	T (°C)	PAR (μmol m^{-2} s^{-1})
T1	8.23	242.5	4.68	24.93	3.7	14.37	97
T2	8.10	191.9	5.39	25.63	4.2	13.35	105
T3	8.11	190.4	5.38	25.04	4.1	13.52	100

ORP: oxido-reduction potential, DO: dissolved oxygen, PAR: Photosynthetically Active Radiation.

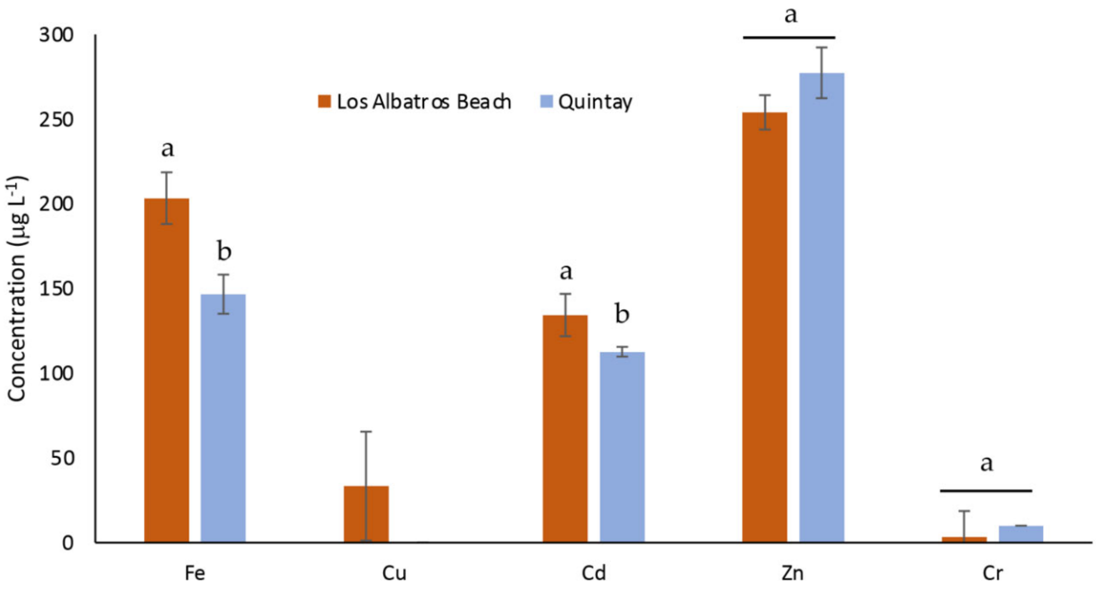

Figure S1. Concentration of some heavy metals studied in the seawater from Los Albatros Beach and Quintay. Bars represent mean ± SD (n= 3). Identical letters indicate no significant difference (P > 0.05). (Limit of detection: Fe = 100 µg L^{-1}, Cu = 40 µg L^{-1}, Cd = 20 µg L^{-1}, Zn = 50 µg L^{-1}, Cr = 10 µg L^{-1}).
